# Nutrition and Lifestyle-Related Factors as Predictors of Muscle Atrophy in Hematological Cancer Patients

**DOI:** 10.3390/nu16020283

**Published:** 2024-01-18

**Authors:** Christiane S. Staxen, Sara E. Andersen, Lars M. Pedersen, Christian B. Poulsen, Jens R. Andersen

**Affiliations:** 1 Department of Hematology, Zealand University Hospital, 4000 Roskilde, Denmark; 2 Department of Nutrition, Exercise and Sports, University of Copenhagen, 1958 Frederiksberg, Denmark; 3 Department of Clinical Medicine, University of Copenhagen, 2200 Copenhagen, Denmark

**Keywords:** cancer, hematology, cytostatic treatment, bioelectrical impedance analysis, muscle mass, nutrition, quality of life, physical activity

## Abstract

Background: Cancer and side effects from cytostatic treatment commonly affect nutritional status manifested as a decrease in muscle mass. We aimed to investigate the impact of nutrition and lifestyle-related factors on muscle mass in patients with hematological cancer. Methods: Dietary intake, food preferences, quality of life (QoL), and physical activity level (PAL) were monitored during 1–2 cytostatic treatment series. Body composition was estimated using bioelectrical impedance analysis (BIA). Results: 61 patients were included. Weight loss and loss of muscle mass were detected in 64% and 59% of the patients, respectively. Muscle mass was significantly positively correlated to increasing PAL (*p* = 0.003), while negatively correlated to increasing age (*p* = 0.03), physical QoL (*p* = 0.007), functional QoL (*p* = 0.05), self-perceived health (*p* = 0.004), and self-perceived QoL (*p* = 0.007). Weight was significantly positively correlated to increased intake of soft drinks (*p* = 0.02) as well as the favoring of bitter grain and cereal products (*p* = 0.03), while negatively correlated to increasing age (*p* = 0.03) and increasing meat intake (*p* = 0.009) Conclusions: Several nutritional and lifestyle-related factors affected change in body composition. The clinical significance of these changes should be investigated in controlled, interventional studies.

## 1. Introduction

Weight loss and especially the loss of muscle mass is frequently seen in cancer patients as a result of cachexia-associated metabolic changes which seem to be mediated by secretion of pro-inflammatory cytokines from cancer cells and from the immune system of the host, including tumor necrosis factor (TNF), interferon-gamma (IFN-γ), and several interleukins (IL-6, IL-1β) [1]. These metabolic changes involve increased proteolysis and lipolysis as the fuel for hepatic gluconeogenesis, mitochondrial dysfunction, and decreased insulin sensitivity [2,3]. Concurrently, loss of muscle mass in patients with hematological cancer is related to poor nutritional status, which is mainly described to be a result of the side effects of cytostatic treatment and/or the cancer disease itself [4,5]. Side effects with consequences for the patients’ nutritional status include nausea, vomiting, changes in the sense of smell and taste, mucositis, decreased energy levels, and intestinal disorders, which can lead to malabsorption, diarrhea, and constipation [6,7,8].

Patients diagnosed with hematological malignancies often have a normal nutritional status at the time of diagnosis [5,9]; however, side effects from cytostatic treatment can adversely affect the nutritional status as treatment progresses [9,10]. Poor nutritional status and loss of muscle mass have been associated with increased chemo-induced toxicity [11,12,13], decreased cytostatic tolerance [14], and delay and/or reduction of cytostatic dosing [15,16], as well as inferior treatment outcome [17,18] and reduced survival rate [19,20,21]. The estimated prevalence of malnutrition and/or risk of malnutrition among different types of cancer patients have been reported to be between 30% and 80%, with the highest prevalence rates in patients with advanced stages of cancer [22,23,24,25,26,27,28].

Several studies assessing different cancer diagnoses have also associated the loss of muscle mass with a decrease in quality of life (QoL) [29,30,31,32,33], with decreased physical functionality [30,34], and with low physical activity level (PAL) [21]. These factors seem to further affect patients’ nutritional status negatively [35,36,37].

A multimodal treatment approach has been shown to reduce the loss of muscle mass in patients with other types of cancer than the hematological [14,38]. The aim of this observational study was to investigate possible associations between energy and protein intake, food preferences, QoL, PAL, and eating and TV habits at main meals, with change of muscle mass as the primary endpoint and weight change as the secondary endpoint in hematologic cancer patients undergoing cytostatic treatment.

## 2. Materials and Methods

### 2.1. Patients

Patients receiving myelosuppressive cytostatic treatment for lymphoproliferative malignancies, acute leukemia, and multiple myeloma in the period 6 April–8 July 2021 at the Department of Hematology, Zealand University Hospital, Roskilde, Denmark, were consecutively enrolled in the study. The treatment took place either on an outpatient basis or at the ward. The inclusion criteria were ongoing myelosuppressive cytostatic treatment for hematological cancer with more than one treatment cycle remaining in their program, >18 years of age, signed informed consent, no need for an interpreter, no conditions which could interfere with their ability to understand the requirements of the study, not pregnant or breastfeeding. The Project was handled in accordance with the Principles of the Helsinki declaration and has been approved by The Research Ethics Committee for Faculties of SCIENCE and SUND (case: 504-0226/20-5000, 7 December 2020) as well as the Regional Scientific Ethics Committee of Zealand (project-ID-no: SJ881, 24 November 2020).

### 2.2. Data Collection

Every patient was followed through 1–2 arbitrary but consecutive treatment cycles, which will be referred to as the follow-up period. Clinical data were collected from medical records. The bioelectrical impedance analysis (BIA) method was used to estimate body composition and was performed on 8-point Seca 515/514^®^ [39]. The BIA was performed immediately before or after the patients’ planned cytostatic treatments. Questionnaire interviews were performed throughout the follow-up period, after the patient’s attendance at the hospital and/or by telephone. 24 h dietary recalls [40] and food preference questionnaire interviews were conducted with two to five days intervals and subsequently quantified using the Danish program for analyzing the content of foods (Vitakost^®^, Kolding, Denmark). In addition, at every second contact, questionnaire interviews about QoL and PAL were conducted. Patients were interviewed regarding their eating and TV habits at main meals at the first interview and at the end of the follow-up period.

### 2.3. Questionnaires

The questionnaires were designed by the investigators by a selection of relevant parts from existing validated questionnaires. Prior to application, all questionnaires were tested on 15 healthy independent individuals of different ages to ensure a high level of understanding. Also, the food preference questionnaire was developed by the investigators, as no relevant pre-designed questionnaires were available. The following categories were included: change in intake, decreased appetite, experience of change in taste, and preferences for meat, fruit and vegetables, dairy products, grain and cereal products and soft drinks. The possible answer options in the questionnaire were categorized on a scale from 1–5 from “not at all” to “very much”.

The questions regarding preferences referred to intake amount before and after initiation of treatment. Preferences for frying and relevant basic flavors were a topic in each category.

The applied QoL questionnaire was designed as an adjustment of the already-existing, validated questionnaires QLQ-C30 (ver. 3) [41] and FACT-Lym (ver. 4) [42]. The scale was copied from FACT-Lym, where all questions in the first four categories were included. Additionally, selected questions regarding side effects were included from QLQ-C30.

The category of physical well-being consisted of thirteen questions, including the most commonly-experienced side effects from cytostatic treatment. The social well-being category consisted of nine questions, the category of emotional well-being consisted of nine questions, and the functional well-being category consisted of eight questions. In addition, the applied adjusted questionnaire finally contained two questions regarding self-assessment of overall health and QoL, identical to the final category in the QLQ-C30 questionnaire.

The PAL questionnaire was designed to estimate change in PAL during cytostatic treatment. The questionnaire contained two questions scaled from 1–5 (“not at all”—“a lot”). The included questions were based on WHO’s GPAQ analysis guide [43].

The questionnaire regarding eating and TV habits at main meals was designed by the investigators in order to estimate how often the patients were eating alone or with others, as well as how often the TV was turned on during meals. The questionnaire contained four questions scaled from 1–7 to indicate how many times a week the given eating circumstance occurred.

### 2.4. Statistical Analysis

All statistical analysis is performed using R (ver. 4.1.1). Descriptive data are presented as number (%) or mean ± SD.

All changes in weight, muscle, and fat mass are defined as weighted values of daily percentage change, depending on individual follow-up period duration. Energy and protein ingestion is measured as the difference between intake and the estimated requirement in %. Variables of food preferences are defined as the average preference during the follow-up period. Intake of food categories were assigned binary values prior to analysis.

Continuous data is analyzed using Pearson correlation analysis, presented by correlation coefficient r and *p*-value. A significance level of *p* ≤ 0.05 is used.

A multiple linear regression analysis is performed to examine the influence of the discrete nutritional variables on the primary and secondary endpoints. Furthermore, a least absolute shrinkage and selection operator (LASSO) regression analysis method is used, aiming at enhancing the prediction accuracy of the statistical model by shrinking the regression coefficients associated with the least important variables to zero. This includes both variable selection and an L1 regularization method. A significance level of *p* ≤ 0.05 is used.

## 3. Results

### 3.1. Baseline Characteristics

The baseline characteristics of the patients included in the study are shown in Table 1. The patients participated a mean of 36 days ± 13 and received a mean of 1.6 treatment cycles ± 0.5. A total of 107 patients with lymphoma, acute leukemia, or multiple myeloma diagnoses were referred for myelosuppressive cytostatic treatment. 41 (38%) patients did not meet the study’s inclusion criteria, while 66 (62%) patients were enrolled in the study. A further four patients were excluded at the patient’s request, while one patient was excluded due to missing BIA data at the end of the follow-up. Therefore, 61 (57%) patients could be included in the final study group available for study analyses.

### 3.2. Body Composition

BIA was performed two (24 patients (39.3%)) or three (37 patients (60.7%)) times in connection with a consecutive series of cytostatic treatment, a mean of 2.6 times ± 0.5. Changes in weight, muscle, and fat mass presented in Table 2 are classified as any measurable changes between the first and last measurement.

### 3.3. Nutritional Intake

24 h dietary recall interviews were performed a mean of 10.8 times ± 3.6 during the follow-up period, 5 times being the lowest number of dietary recalls and 17 times being the highest. Two patients (3%) met 100% of their estimated energy requirement, while 28 patients (46%) met 75%. One patient (1.6%) met 100% of their estimated protein requirement, while 22 patients (36%) met 75%.

79% of the patients experienced a decrease in appetite at some point during the follow-up period. The mean reduction in appetite score compared with the appetite score before the initiation of cytostatic treatment was 1.8 on the 1–5 scale (“not at all”–“a lot”). 77% of the patients experienced changes in flavor perception during the follow-up period, distributed over a total of 193 times.

No statistically significant correlations were found between change in either muscle mass nor weight and energy intake, protein intake, decreased appetite, or experience of residual flavor (Table 3).

Patients were asked about their intake of different food categories before and after the initiation of cytostatic treatment a mean of 10.8 times ± 3.6. The average intake of meat, fruit and vegetables, dairy, and grain and cereal products decreased after the initiation of cytostatic treatment, while the average intake of soft drinks increased (Table 4).

No statistically significant correlations were found between change in muscle mass and any preferences, while a statistically significant negative correlation was found between change in weight and preference for bitter grain and cereal products (*p* = 0.03) (Table 5).

A statistically significant negative correlation was found between weight change and increase in meat intake (*p* = 0.009), while a statistically significant positive correlation was found between weight change and increased consumption of soft drinks (*p* = 0.02) (Table 5).

### 3.4. QoL, Physical Activity and Eating Situations

Patients were asked about their QoL and PAL a mean of 5.8 times ± 1.7, while they were asked about their eating habits a mean of 2 times ± 0.

The highest average decrease in QoL within the investigated parameters was found in the following order (mean ± SD): functional well-being (1.94 ± 0.59) > physical well-being (1.81 ± 0.42) > emotional well-being (1.44 ± 0.29) > social well-being (1.30 ± 0.39). Correlations are presented in Table 6.

Due to the reversed scale of self-perceived health and QoL, this suggests that all parameters of decreased QoL and low self-perceived health and QoL seem to have affected the change in muscle mass negatively while no categories affected change in weight.

The average score of PAL was 3.48. A weak statistically significant positive correlation was found between PAL and change in muscle mass (*p* = 0.003). This suggests that increased levels of physical activity seem to have a positive effect on the change in muscle mass.

No statistically significant correlations were found between eating and TV habits at main meals and change in neither muscle mass nor weight.

### 3.5. LASSO Regression Analysis

LASSO regressions were generated with change in muscle mass and weight in percent as dependent variables, with the purpose of attaining the subset of predictors that reduces the prediction error for the measurable dependent variable and thereby increases model accuracy and model interpretability. For the training and test of the model, an 80/20 split of data was used. After selection in the LASSO model, no independent variables were found relevant for change in muscle mass or weight for further hypothesis testing.

## 4. Discussion

The main findings of this study are that PAL and QoL have a positive effect on change in muscle mass during cytostatic treatment. On the contrary, our data suggest that diet adjustments and food preferences have less impact on the change of muscle mass and weight, while eating and TV habits seem to have no impact at all.

A decrease in muscle mass and weight is widely seen in cancer patients undergoing chemotherapeutic treatment [33,44,45,46,47], which aligns with the distribution of body mass development in the present study, since the majority of the subjects had a decrease in both muscle mass (59.0%) and weight (62.9%) concurrently to an increase in fat mass (55.7%). The beneficial effect of physical activity on the maintenance of muscle mass in hematological cancer patients undergoing cytostatic treatment has been reported in previous studies [48,49]. Therefore, the results in the present study were expected. One study found that physical activity in combination with a high protein intake over 12 weeks in hematological cancer patients [50]. At the same time, they found that a low level of physical activity combined with a high intake of protein led to a decrease in muscle mass and increased fat mass, which supports the result seen in the present study, that physical activity might affect body composition more than nutritional intake. Physical activity is also documented to reduce fatigue [51,52] and improve both handgrip force and functional mobility in hematologic cancer patients [52]. In addition, a higher level of physical activity appears to have beneficial effects in terms of improving sleep, both quality and duration, which is seen to affect QoL [53]. An increased level of sleep-mediating cytokines, such as IL-6, TNF-α, and sTNF-R in cancer patients could cause impaired sleep quality [54].

A change in muscle mass was in the present study affected by the categories physical and functional well-being as well as self-perceived health and QoL or vice-versa. This correlation has also been reported in similar studies of a wide range of cancers [31,32,33,55], but not in all [56]. Concurrently, QoL is reported to be consistently higher for patients who have high PAL. The largest decrease of QoL, which was found in the categories of functional and physical well-being, is equivalent to the results found in a study where the EORTC QLQ-C30 questionnaire is used as well [57].

Increasing age seems to have a negative effect on change in both muscle mass and weight, which is to be expected. Concurrently, the results show that the five youngest patients all experienced an increase in weight, which had a marked impact on the result. If they were to be considered as outliers and removed from the analysis, the correlation with age would no longer be statistically significant. Conventionally, sarcopenia is caused by many factors, such as decrease in PAL, loss of neuromuscular junctions, changes in hormones, and chronic diseases with inflammation [58,59]. Muscle mass and muscle strength is expected to decrease with age [60], but the presence of severe disease seems to change that.

The dietary composition as well as the investigated food preferences do not seem to have as important an effect on maintenance of muscle mass and weight as expected. However, the results have been obtained from a narrow amount of data due to very small subgroups.

In the present study, the majority did not meet their energy or protein requirements. It is well documented that low energy and protein intake leads to loss of both muscle mass and weight, normally explained by an increase in demands in cancer patients [26,61,62,63]. Nevertheless, this cannot be confirmed in the present study. The results could possibly have aligned more with those of the former-mentioned studies if the follow-up period had been longer, since larger fluctuations in muscle mass and weight may not be expected short term. In one study, it is suggested that meeting the minimum energy recommendations (25 kcal/kg/day) may not be sufficient to attenuate loss of muscle mass in head and neck cancer patients [64]. On the contrary, it has been demonstrated that an intake of protein at 1.2 g/kg/day in hematological cancer patients had a positive impact on muscle mass changes [50]. The recommendation of 1.2–1.5 g/kg/day is based on such studies [65], but other studies in cancer patients did not disclose this effect, not even in patients in remission [66]. The present results could not confirm the recommendation either and explanations could be several. Besides the already-mentioned short follow-up period, the patients might have had a relatively good nutritional status when enrolled in our study, which could be explained by the national treatment guarantee giving patients a legal right to start treatment within 4 weeks after the diagnosis has been established in all malignant diseases.

A decreased appetite and taste alterations are well known side effects from cytostatic treatment [28,67] but the present results do not support these findings, neither concerning decreased appetite nor residual flavor, and no correlations between decreases in either muscle mass nor weight was found, in contradiction to other reported results in similar patient groups [68] and in patients with lung cancer [69]. There are some challenges in our method regarding the estimations of decreased appetite and the presence of residual flavor since the construction of the scales exclusively made it possible to measure respectively the absence of decreased appetite and the presence of residual flavor. On the opposite. It was not possible to measure increased appetite or improved taste because it was estimated from a “one-way” scale. Results might have aligned with the previous mentioned studies.

Considering the study’s strengths, worth mentioning are the close monitoring and the insurance of data validity, as well as statistical analysis encompassing confounders. The repeated use of the 24 h dietary recall method, including intake at both weekdays and weekend days, decreases the risk of errors due to day-to-day variation [70]. A study comparing the 24 h dietary recall method with the doubly-labeled water method, found three 24 h dietary recalls to be sufficient when estimating energy intake [71]. They did not find additional recalls to improve the estimation further. In spite of that, is must be reasonable to assume that day-to-day variations have been eliminated to a satisfying extent in the present study.

The study design has some limitations as well, the short follow-up period being the most obvious, which also to some degree counteracts the close monitoring. This results in a fragmented understanding of the challenges that the patients may face, which mean that important confounders might be overlooked. Furthermore, the size of the study cohort limits the opportunity to achieve an adequate understanding of the relationship between nutritional intake, QoL, physical activity, and muscle atrophy. A combination of a larger study cohort and a prolonged follow-up period might have allowed the methodical strengths of this study to be more prominent.

There was a potential heterogeneity within the patient group in terms of both diagnosis, treatment regimens of different intensity, varying duration between initiation of chemotherapy and monitoring of the patient, and different degrees of response to the cytostatic treatment with a risk that these may constitute confounding variables. However, the size of the study cohort does not enable any analysis of the possible influence of the individual factors. Further larger, controlled studies with a homogeneous patient cohort and a longitudinal design with longer monitoring are therefore warranted.

## 5. Conclusions

We have shown that nutritional and lifestyle-related factors affected the change in weight and muscle mass in patients with hematological cancer. The effect on muscle mass and weight was most evident in relation to physical activity, while dietary components seem to have less importance. Our study needs further validation in larger studies, including associations with clinically relevant variables such as treatment response and long-term outcome of the malignancy. Intervention studies investigating the impact of programs for dietary measures and physical activity on treatment toxicity, response, survival and QoL are also justified.

## Figures and Tables

**Table 1:** Basic characteristics of the 61 included patients. Values are presented as number (%) or mean ± SD.

Characteristics	Patients, n (%)/Mean ± SD
Sex	
Men	40 (65.6%)
Women	21 (34.4%)
Age (years)	66 ± 13
Diagnose	
Lymphoma	56 (91.8%)
Acute leukemia	3 (4.9%)
Multiple myeloma	2 (3.2%)
Number of cytostatic treatments before inclusion	2.4 ± 1.4
Treatment regime	
(R)-CHOP (+like) ^1^	34 (55.7%)
(R)-Benda (+like) ^2^	17 (27.9%)
ABVD (+like) ^3^	5 (8.2%)
G-PEBEN ^4^	1 (1.6%)
CY-VEL-DEX ^5^	2 (3.3%)
DA 3 + 10 ^6^	2 (3.3%)
Complete remission after end of full course of treatment	51 (84%)
BMI at inclusion	
18.5–24.9	21 (34.4%)
25–29.9	28 (45.9%)
>30	12 (19.7%)

^1^ (Rituximab), cyclophosphamide, doxorubicin hydrochloride, vincristine sulfate, prednisone; ^2^ (Rituximab), bendamustine; ^3^ Doxorubicin hydrochloride, bleomycin sulfate, vinblastine sulfate, dacarbazine; ^4^ Pixantrone, etoposide, bendamustine; ^5^ Cyclophosphamide, bortezomib, dexamethasone; ^6^ Daunorubicin, Ara-C/Cytarabin.

**Table 2:** Overview of the number (%) of patients with change in muscle and fat mass and weight during the study period, as well as mean ± SD of daily percentage change during the follow-up period, compared to the first measurement.

	Muscle Mass	Fat Mass	Weight
Decrease	36 (59.0)	26 (42.6)	39 (63.9)
Increase	23 (37.7)	34 (55.7)	22 (36.1)
No change	2 (3.3)	1 (1.6)	0
Daily percentage change	−0.07 ± 0.25	0.05 ± 0.44	−0.03 ± 0.13

Pearson correlation analysis showed statistically significant negative correlations between both age and change in weight (*p* = 0.03) as well as age and change in muscle mass (*p* = 0.03).

**Table 3:** Results from Pearson correlation analysis between change in weight and change in muscle mass, and energy intake, protein intake, decreased appetite and experience of residual flavor. Comparing before and end of the follow-up period.

Variables	Muscle Mass		Weight	
	Pearson’s r	*p*-Value	Pearson’s r	*p*-Value
Energy intake	0.09	0.48	0.12	0.34
Protein intake	0.16	0.21	0.1	0.15
Decreased appetite	−0.18	0.15	−0.14	0.27
Experience of residual flavor	−0.14	0.30	−0.08	0.56

**Table 4:** Average percentage distribution in intake for the individual food categories after the start of cytostatic treatment compared to intake before the initiation of cytostatic treatment for respectively the patients who lost muscle mass and the patients who did not during the follow-up period.

Variables	Decrease in Muscle Mass			No Decrease in Muscle Mass		
Intake	Decreased (%)	Unchanged (%)	Increased (%)	Decreased (%)	Unchanged (%)	Increased (%)
Meat	70.3	21.6	8.1	60.9	13.0	26.1
Fruit and vegetables	57.9	10.5	31.6	60.9	21.7	17.4
Dairy	52.6	29.0	18.4	65.2	13.1	21.7
Grain and cereal	63.2	26.3	10.5	52.2	34.8	13.0
Soft drinks	44.7	21.1	34.2	34.8	13.0	52.2

**Table 5:** Correlations from multiple linear regression analysis between change in muscle mass and weight and increase in intake of included food categories compared with the intake before initiation of cytostatic treatment, as well as preferences for different flavor nuances.

Variables	Muscle Mass		Weight	
	Estimate	*p*-Value	Estimate	*p*-Value
Increase in intake				
Meat	−0.09	0.54	−0.18	0.009
Fruit and vegetables	−0.07	0.47	0.01	0.74
Dairy	0.01	0.90	0.03	0.49
Grain and cereal	0.17	0.20	0.08	0.15
Soft drinks	0.04	0.60	0.07	0.02
Meat preference				
Red meat	0.18	0.33	−0.002	0.98
Dark meat	0.20	0.07	0.02	0.69
Light meat	0.07	0.42	0.05	0.20
Fruit and vegetables preferences				
Sweet	−0.01	0.90	−0.07	0.15
Sour	−0.01	0.89	0.006	0.82
Bitter	0.05	0.31	0.01	0.68
Dairy preferences				
Sweet	−0.03	0.45	−0.02	0.63
Sour	0.03	0.49	0.001	0.95
Salty	−0.02	0.67	0.006	0.78
Umami	0.004	0.94	−0.008	0.70
Grain and cereal preferences				
Sweet	−0.01	0.81	0.02	0.44
Sour	−0.01	0.74	−0.001	0.95
Bitter	0.03	0.55	0.05	0.03
Soft drink preferences				
Sweet	−0.0009	0.99	−0.01	0.66
Sour	0.03	0.58	0.002	0.94
Bitter	−0.01	0.85	0.006	0.72

**Table 6:** Results from Pearson correlation analysis between change in weight and change in muscle mass and the variables of QoL, PAL, and eating and TV habits at main meals.

Variables	Muscle Mass		Weight	
	Pearson’s r	*p*-Value	Pearson’s r	*p*-Value
QoL				
Physical	−0.34	0.007	−0.05	0.70
Social	−0.25	0.05	0.10	0.43
Emotional	−0.23	0.08	−0.15	0.26
Functional	−0.26	0.05	0.01	0.92
Self-perceived health ^1^	0.37	0.004	0.21	0.10
Self-perceived QoL ^1^	0.34	0.007	0.17	0.19
Physical activity				
PAL	0.37	0.003	0.17	0.18
Eating and TV-habits				
Eating with others	0.05	0.72	0.04	0.78
Eating alone	−0.02	0.90	−0.21	0.11
TV on with others	−0.06	0.66	0.008	0.95
TV on alone	0.08	0.54	−0.06	0.64

^1^ The scale of self-perceived health and QoL were reversed compared to scales on QoL.

## Data Availability

The data presented in this study are available on request from the corresponding author on reasonable request.
